# Genetic Modulation of the GLUT1 Transporter Expression—Potential Relevance in Complex Diseases

**DOI:** 10.3390/biology11111669

**Published:** 2022-11-16

**Authors:** Anna Kulin, Nóra Kucsma, Balázs Bohár, Botond Literáti-Nagy, László Korányi, Judit Cserepes, Anikó Somogyi, Balázs Sarkadi, Edit Szabó, György Várady

**Affiliations:** 1 Doctoral School of Molecular Medicine, Semmelweis University, 1085 Budapest, Hungary; 2 Institute of Enzymology, Research Centre for Natural Sciences, 1117 Budapest, Hungary; 3 Doctoral School of Biology, Eötvös Loránd University, 1117 Budapest, Hungary; 4 Drug Research Center, 8230 Balatonfüred, Hungary; 5 CellPharma Kft, 1119 Budapest, Hungary; 6 2nd Department of Internal Medicine, Semmelweis University, 1088 Budapest, Hungary

**Keywords:** glucose transporter, single nucleotide polymorphism, transcription factor binding sites, flow cytometry, luciferase expression assay

## Abstract

**Simple Summary:**

The human GLUT1 membrane protein is the key glucose transporter in numerous cell types and the expression level of this protein has a role in several diseases, including cancer or Alzheimer’s disease. In this work, we measured the expression level of this protein and analyzed the genetic and regulatory background of the alterations. Our results suggest that some genetic variations can modulate the amount of GLUT1. Besides, regulatory factors such as glucose and hypoxia also showed some effect on the outcome. These results should contribute to a more detailed understanding of the background of membrane protein expression and its potential role in associated diseases.

**Abstract:**

The human GLUT1 (SLC2A1) membrane protein is the key glucose transporter in numerous cell types, including red cells, kidney, and blood-brain barrier cells. The expression level of this protein has a role in several diseases, including cancer and Alzheimer’s disease. In this work, to investigate a potential genetic modulation of the GLUT1 expression level, the protein level was measured in red cell membranes by flow cytometry, and the genetic background was analyzed by qPCR and luciferase assays. We found significant associations between red cell GLUT1 levels and four single nucleotide polymorphisms (SNP) in the coding SLC2A1 gene, that in individuals with the minor alleles of rs841848, rs1385129, and rs11537641 had increased, while those having the variant rs841847 had decreased erythrocyte GLUT1 levels. In the luciferase reporter studies performed in HEK-293T and HepG2 cells, a similar SNP-dependent modulation was observed, and lower glucose, serum, and hypoxic condition had variable, cell- and SNP-specific effects on luciferase expression. These results should contribute to a more detailed understanding of the genetic background of membrane GLUT1 expression and its potential role in associated diseases.

## 1. Introduction

**Figure 1:**
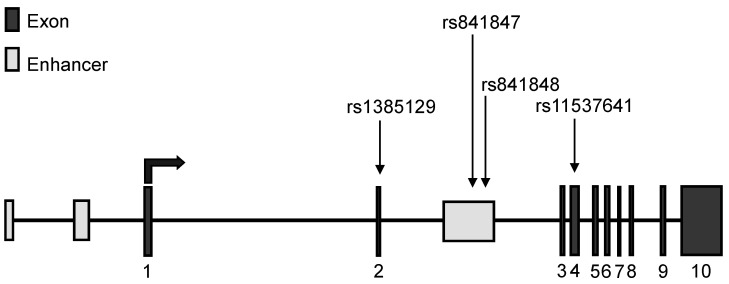
Structure of human SLC2A1 gene with the 10 exons (indicated as 1–10) and the putative enhancers [5]. The 4 examined SNPs are also presented.

GLUT1, the main glucose transporter of the red blood cell, has broad substrate specificity for pentoses and hexoses, including vitamin C [1], and it facilitates glucose transport in many cell types, including the endothelial cells of the blood-brain barrier (BBB) or the kidney cortex mesangial cells [2,3]. The highly conserved GLUT1 contains 492 amino acids, forming a glycosylated protein of about 55 kDa [4,5]. This protein is encoded by the SLC2A1 (solute carrier family 2-facilitated glucose transporter member 1) gene, located in the 34.2 position of the p arm of chromosome 1. The gene includes 33,802 base pairs, with three putative enhancer regions and 10 exons [6] (see Figure 1).

Studies on GLUT1 have shown an association between the expression of this protein andseveral disease conditions. While missense or nonsense mutations in the SLC2A1 gene are very rare, regulatory alterations in its expression are often found in major human diseases. Since GLUT1 mediates glucose transport into the brain at the BBB, defects in the protein can cause serious problems in the nervous system. Mutations in the SLC2A1 gene cause glucose transporter type 1 deficiency syndrome, which limits brain glucose availability [7]. This condition can induce epilepsy, movement disorders, and cognitive impairment [8]. Reduced GLUT1 expression at the BBB was found to be associated with Alzheimer’s disease, characterized by decreased glucose transport in the early stage of the disease [9,10]. Although the glucose transporter most involved in type two diabetes mellitus (T2DM) is the insulin-modulated GLUT4 protein, there are some GWAS data concerning the polymorphisms of the SLC2A1 gene related to T2DM (https://t2d.hugeamp.org/ (2020)) [11]. Besides, serious complications of this disease (e.g., diabetic nephropathy and diabetic retinopathy) are also associated with some SLC2A1 polymorphisms, including rs1385129, rs841847, and rs841848 [12,13,14], which SNPs examined in this study. It should also be noted that in various tumor cells, especially under hypoxic conditions, elevated glucose metabolism is present and the overexpressed GLUT1 transporter has an important role in the modulation of this process [15,16,17]. Thus, regulatory GLUT1 alterations, based on genetic background, may affect a wide range of metabolic alterations and disease conditions.

The four SNPs in the SLC2A1 gene examined here (Figure 1) have minor allele frequencies between 0.19–0.25 in Europe, thus representing relatively large populations. One of the most studied polymorphisms is rs1385129, which was found to increase the incidence of diabetic nephropathy in different cohorts (Tunisian [12], and Kurdish [13]). This synonymous SNP is a member of a large haplotype block (Supplementary Figure S1) located in exon 2. The SNPs rs841848 and rs841847 are in the 2nd intron, in a putative enhancer (enh-2) region, at 164 bp from each other. Interestingly, these SNPs were also found to increase the risk of diabetic nephropathy [12,18]. While variant rs841848 is a part of a relatively large haplotype block (Supplementary Figure S1), rs841847 is independent of this haplotype (it is not in linkage disequilibrium with rs841848, see Supplementary Table S1). In addition, based on our own sequencing results (Supplementary Table S2), variant rs11537641, located in exon 4 (synonymous), was also included in the study.

In order to explore the genetic and regulatory background of GLUT1 expression, potentially related to various diseases, first we have studied the expression level of this protein in the human red blood cell membrane. As described earlier, a quantitative estimation of the erythrocyte membrane proteins can be achieved by using our antibody-based flow cytometry technology, which allows for exploring the genetic background of the alterations in membrane protein expression and the connected disease conditions [19,20,21]. We have documented that by using this erythrocyte ghost-forming method and applying well-established, specific monoclonal antibodies—in contrast to other technologies (e.g., Western blotting or direct chemical labeling)—even small differences in the red cell expression levels can be properly quantitated. Using these results of the expression level of GLUT1, we performed sequencing in samples with low, medium, and high expression levels. With this step, we were able to choose the SNPs that show potential association with the amount of GLUT1. To make these results statistically relevant, we used a genotyping method in the whole examined population.

Since the red cell expression data reflect the role of the genetic haplotypes, we analyzed the actual effect of the variants with luciferase reporter assays. Two different human cell lines were used in these studies, the immortalized human embryonic kidney derived (HEK) cells, which may reflect some of the kidney-type regulatory features, and a hepatoma cell line (HepG2) which may be informative for a more complex, hepatocyte-like regulatory mechanism. We have used the SNP-containing luciferase reporter constructs with a minimal promoter and measured the basic enhancer or suppressor effects of the selected SNP variants. In addition, in the metabolically more relevant HepG2 cells, we have also examined the potential GLUT1 regulatory role of these polymorphisms by reducing glucose and oxygen levels or by adding insulin into the medium with reduced serum components. The direct exploration of tissue-dependent effects of specific SNPs provided here may significantly extend the data obtained from previous case-control studies and focus attention on the role of GLUT1 regulation in relevant metabolic or disease conditions.

## 2. Materials and Methods

### 2.1. Samples and Laboratory Data

This study was conducted involving 207 Hungarian individuals (87 males and 120 females) with an average age of 60 (±13). 120 samples were obtained from type two diabetic (T2DM) patients, 59 samples from age-matched controls, and 28 samples from healthy volunteers. Written informed consent was obtained from all patients, and the study was approved by the Scientific and Research Committee of the Medical Research Council, Hungary (ETT TUKEB references: 19680-3/2019/EKU, 2367-1/2019/EKU). All methods were performed in accordance with the relevant guidelines and regulations. The samples were obtained from the Drug Research Center (DRC, Balatonfüred, Hungary) and the 2nd Department of Internal Medicine, Semmelweis University (SE, Budapest, Hungary). Besides, to increase the sample size we also analyzed samples from in-house volunteers. The disease-related laboratory data (HbA1c, glucose, and insulin levels, HOMA indices, RBC and WBC, etc.) and anthropometric data (weight, height, BMI, and waist circumference) of the participating patients were provided by the two collaborators, however, there were no such data for the in-house volunteers. In addition, we received information about the disease-related complications of 23 cases (albuminuria (*n* = 16), neuropathy (*n* = 11), nephropathy (*n* = 7) and retinopathy (*n* = 4)), the family medical history (hypertension, obesity, gout, etc.) and the medication parameters. The clinical diagnosis of T2DM was established according to the criteria of the American Diabetes Association (ADA) [22].

### 2.2. Flow Cytometry

The expression level of the RBC membrane proteins was measured by a method developed by our research group (for details, see refs [10,23]). The RBC membranes were fixed with 1% formaldehyde solution and the resulting RBC membranes (ghosts) were labeled by Alexa Fluor 647 (AF647) conjugated wheat germ agglutinin (WGA-AF647, Thermo Fischer Scientific, Waltham, MA, USA, W32466). Then the samples were labeled with GLUT1 rabbit monoclonal antibody (Abcam, Cambridge, UK, ab115730), reacting with an intracellular epitope, followed by a secondary (Alexa Fluor 488-labeled goat anti-rabbit (H + L) antibody, Thermo Fischer Scientific, Waltham, MA, USA, A-11008). RBC ghosts were analyzed for antibody staining by Attune NxT acoustic flow cytometer (Applied Biosystems, Life Technologies, Carlsbad, CA, USA) with excitation wavelengths: 488 nm, emission filters: 530/30 nm for AF488 and excitation wavelengths: 637 nm, emission filters: 670/14, for AF647. The primary and secondary antibodies were titrated to provide maximum labeling.

### 2.3. SNP Selection and Genetic Analysis

Genomic DNA was isolated from whole blood using Gentra Puregene Blood Kit (Qiagen, Germantown MD, USA, 158467) according to the manufacturer’s protocol. For DNA sequencing, we designed primers for all the exons in the SLC2A1 gene (Supplementary Table S3). After amplifying, the PCR products were run in a 1 *w*/*v*% agarose gel and purified by using Wizard SV Gel (Promega, Madison, WI, USA) and PCR Clean-Up System (Promega, Madison, WI, USA, A9281). The tested SNPs (Table 1) were selected based on sequencing and the previous literature data. Genotyping experiments were carried out with TaqMan-based qPCR analysis (Applied Biosystems, Life Technologies, Carlsbad, CA, USA, Software v2.3), and were performed by a StepOnePlus device (Applied Biosystems, Life Technologies, Carlsbad, CA, USA) with premade assay mixes (Thermo Fischer Scientific, Waltham, MA, USA) and master mix (Thermo Fischer Scientific, Waltham, MA, USA, 4371355). The specificity of the probes was verified by sequencing. These experiments, together with flow cytometric analysis, provided information on the effect of the tested SNPs and the related haplotype. 

### 2.4. Reporter Vector Constructions

To generate recombinant luciferase vector constructs, we used a modified (mini-TK promoter vector previously designed by Boglárka Zámbó) basic pGL3 vector (Promega, Madison, WI, USA, E1751). In this experiment, we analyzed inserts that contained the minor allele only for the tested SNP (for insert sizes and primers see Supplementary Table S4). The DNA fragments of interest were amplified by PCR and were inserted in the mini-TK promoter vector by using T4 ligase (Thermo Fischer Scientific, Waltham, MA, USA, EL0011), after digestion by NheI (NEB, Ipswich, MA, USA, R0131S) and XhoI (NEB, Ipswich, MA, USA, R0146S) restriction enzymes (Supplementary Figure S2). The resulting constructs were transformed into *E. coli* Dh5α ultra-competent cells and purified using the PureYield Plasmid Miniprep System (Promega, Madison, WI, USA, A1223). To ensure the ligation was correct, we sent the constructs for Sanger sequencing.

### 2.5. Cell Culture, Transfection, and Treatments

For the cellular experiments, we used human embryonic kidney 293T cells (HEK-293T) and human hepatoma cells (HepG2—frequently used in diabetes-related studies), which were mycoplasma-free cell lines. HEK-293T and HepG2 cells were grown in DMEM/high glucose/GlutaMAX medium (Gibco, Thermo Fischer Scientific, Waltham, MA, USA, 31966-021) completed with 10% heat-inactivated fetal bovine serum (FBS, Gibco, Thermo Fischer Scientific, Waltham, MA, USA, 10500-064) and 0.1% gentamicin (Gibco, Thermo Fischer Scientific, Waltham, MA, USA, 15710-49) at 37 °C and 5% CO_2_.

### 2.6. Dual Luciferase Assay

To determine the potential expression regulatory effects, including the potential enhancer/suppressor activity of the surrounding region, we cloned their respective wild-type and mutant regions into the pGL3 vector (see Supplementary Figure S2) and measured luciferase activity in the transfected HEK-293T and HepG2 cells. For the reporter assay, 40,000 cells/well were plated in 48-well plates both for HEK-293T and HepG2 cells. For transfection, 450 ng of pGL3 TK-mini (vector with thymidine kinase promoter) constructs were used together with 50 ng pRL-TK (Renilla luciferase vector with HSV-Thymidin kinase promoter, Supplementary Figure S2) plasmid. The transfection of HEK-293T cells was carried out with Turbofect reagent (Thermo Fischer Scientific, Waltham, MA, USA, R0531) while HepG2 was transfected by using JetOptimus (Polyplus, Berkeley, CA, USA, 117-07).

For examining the regulatory effects of various incubation conditions, HepG2 liver-related cells, potentially more relevant to metabolic diseases, were treated for 24 h after transfection. For treatments with glucose and insulin, using FBS free medium was necessary, because the exact composition of FBS is not known, and it is conceivable that it contains these compounds. For testing the effect of glucose concentration, we exchanged the medium for an FBS- and glucose-free medium (Gibco, Thermo Fischer Scientific, Waltham, MA, USA, 11966-025). For insulin treatment, we used a medium without FBS and a low amount (1 g/L) of glucose (Gibco, Thermo Fischer Scientific, Waltham, MA, USA, 11885-084), then, 6 h before the measurements, 100 nM insulin (Sigma, Darmstat, Germany, I0516) was added to the samples. This experiment setting was based on the previous literature data. The generally used insulin amount is 100 nM and usually, cells are starved for 24 h before measurements [24,25]. For hypoxia treatment, cells were placed in a humidified, hypoxic incubator (37 °C), flushed with a gas mixture of 5% CO_2_, 92% N_2,_ and 3% O_2_. Physiological hypoxia is likely to be in the range of 2–6% oxygen [26]. This suggests that with 3% of oxygen, the hypoxia response elements may well upregulate, and this condition is also acceptable for the cells.

For the analysis, we used a Dual Luciferase Reporter Assay System kit (Promega, Madison, WI, USA, E1910), according to the manufacturer’s guidelines. Luciferase reaction was measured 48 h after transfection, with VICTOR X3 Multilabel Plate Reader (Perkin Elmer, Waltham, MA, USA). Firefly luciferase activity was normalized to Renilla luciferase activity in all experiments. Each DNA fragment containing the specific SNPs and the surrounding regions was tested in triplicate, and at least three independent experiments were performed.

### 2.7. Bioinformatic Analysis

In order to predict transcription factor binding sites (TFBS) affected by the examined SNPs, we used the matrix scan function of the Regulatory Sequence Analysis Tools (RSAT, http://rsat.sb-roscoff.fr/index.php (2022)). With this tool, we can predict and analyze the transcription factor binding sites. For the usage of this tool, on the one hand, we had to filter the 100 nucleotide long regions around the SNPs (Single Nucleotide Polymorphism)—upstream and downstream. For each SNP, we had two different sequences. First, that which contains the original/reference sequence (with the reference allele of the SNP in the middle of it) and the other, which contains the alternative sequence (with the alternative allele of the SNP in the middle of it). On the other hand, we downloaded the newest version of the position weight matrices (PWM) of the transcription factors (TF) from the Jaspar database (http://jaspar.genereg.net (2022)). This matrix contains the representation of different motifs or patterns in biological sequences. A position weight matrix (PWM) is a model for the binding specificity of a TF and can be used to scan a sequence for the presence of DNA words that are significantly more similar to the PWM than to the background. With our sequences and with the position weight matrix, we could predict the binding sites of different transcription factors in the region around the SNPs, both for the reference sequences and for the sequences, which are containing the SNPs’ alternative alleles. The matrix scan module can calculate a *p*-value for each binding site (referring to the probability of binding the given sequence) and we worked with only those, which was less than 0.0005 (*p* < 0.0005). After the matrix scan module calculated the TFBSs (Transcription Factor Binding Sites), we had two different results. The first contains only those TFBSs which came from the reference sequences with the reference allele of the SNP, and the other part, which contains only those TFBSs which came from sequences with the SNPs’ alternative alleles in them. Then we compared the differences between the two results, and we kept only those TFBSs which were unique. This means that we worked with only those TFBSs, which were only in one of the results (came from the reference or came from the alternative sequences).

### 2.8. Statistical Analyses

Statistical analyses were performed using the Prism 8.0.1 software (GraphPad, San Diego, CA, USA). To compare genotypes and protein levels, we chose the Kruskal–Wallis test with a Dunn post-hoc test. The potential association between the expression levels of GLUT1 and the occurrence of the disease was analyzed with the Mann–Whitney test. The distribution of the SNPs for all variants in the examined samples conformed to the Hardy–Weinberg equilibrium, examined by the χ^2^ test. 

All dual-luciferase experiments were conducted at least three times, and each of these measurements included three replicates. To compare relative luciferase activities between the constructs with different alleles, we used Welch’s *t*-test. In the statistical analyses, *p* < 0.05 was considered a statistically significant difference.

## 3. Results

We performed statistical analysis of the main anthropometric and laboratory data. As expected, main diabetes-specific data (BMI, HbA1c levels, fasting glucose, insulin level, and HOMA-index, etc.) differed significantly between the control and T2DM groups (see Supplementary Table S5).

In our studies, we first examined the potential differences in the red cell GLUT1 expression levels in control and diabetic patients, respectively (Supplementary Figure S3). Besides, we also analyzed the expression level in male and female samples separately (Supplementary Figure S4). The reliable recognition of the GLUT1 protein in the red cell membrane was achieved by a well-established commercially available, selective monoclonal antibody (see refs [27,28,29]). Since the variability of the individual GLUT1 expression levels was relatively high, and there was no significant difference in the mean values of the red cell membrane GLUT1 levels in these relatively small cohorts, in order to find statistically relevant SNP-dependent differences, in our further studies we used the combined values of the expression levels measured in the control and T2DM patients.

**Figure 2:**
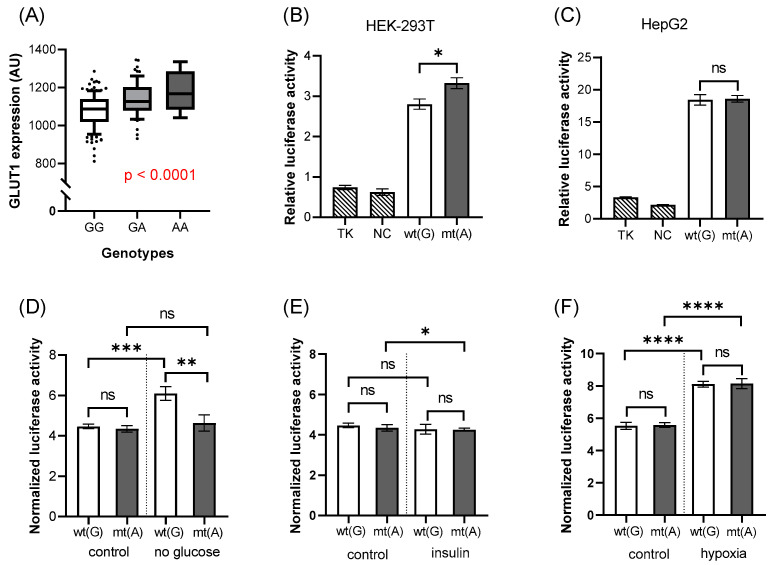
The effects of rs1385129 on GLUT1 expression and luciferase activity. The association between the expression of GLUT1 membrane protein and the genotypes of the variant (**A**). Each boxplot represents samples with different genotypes, in the range of 10–90 percentile, while dots represent samples out of range. Values are expressed as means ± SD. This insert has increased luciferase activity in HEK-293T (**B**) and HepG2 (**C**–**F**) cells compared to the empty vector, and the mutant allele increases this effect in HEK-293T cells (**B**). Wild-type allele has a stimulating effect on luciferase expression under low concentrations of glucose. Insulin has no effect on this region, while hypoxia increases luciferase expression, regardless of the alleles. The *p*-values of the luciferase experiments (**B**–**F**) were calculated using the Welch *t*-test. The stars represent significance at different levels: * *p* < 0.05, ** *p* < 0.01, *** *p* < 0.001, **** *p* < 0.0001. In the case of the treatments, data were normalized to the results of the “empty” vector. wt(G): wild-type allele; mt(A): mutant allele; TK: “empty” vector without insert; NC: negative control (random sequence); ns: not significant; AU: arbitrary unit.

The rs1385129 synonymous variant is located in exon 2 of the SLC2A1 gene. According to the measurements of the red cell membrane expression, the mean level of the GLUT1 membrane protein was significantly higher in the presence of the mutant (A) allele than in the case of the wild-type allele (Figure 2A, *p* < 0.0001). 

The luciferase reporter assay carried out with the rs1385129 polymorphism in both HEK-293T and HepG2 cells revealed that the DNA regions—either with or without the presence of the mutant alleles—had higher luciferase activity compared to the empty vector or to the vector containing a random DNA sequence of the same size (negative control (NC)). Thus, the selected sequence is indeed an enhancer/promoter region within exon 2 of the SLC2A1 gene. As to the effect of the rs1385129 SNP in this region, in the HEK-293T cells, the mutant allele increased luciferase activity more strongly than the wild-type allele (*p* = 0.012, Figure 2B). However, in HepG2 cells (Figure 2C) there was no such difference between the effects of the two alleles. 

As to the effects of various incubation conditions, examined in HepG2 cells, under low glucose incubation conditions (Figure 2D), the presence of the wild-type allele had a stimulating effect on luciferase expression, which was absent in the construct containing the mutant rs1385129 allele. The effect of insulin (Figure 2E) was not appreciable on luciferase activity, while under hypoxic conditions (Figure 2F), luciferase expression significantly increased, independent of the presence or absence of the SNP variant.

**Figure 3:**
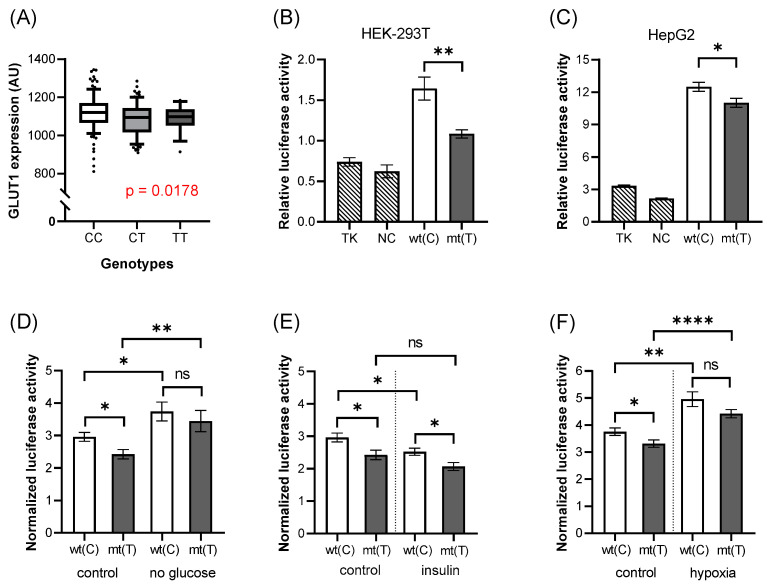
The effects of rs841847 on GLUT1 expression and luciferase activity. The association between the expression of the GLUT1 membrane protein and the genotypes of the variant (**A**). Each boxplot represents samples with different genotypes, in the range of 10–90 percentile, while dots represent samples out of range. This insert has increased luciferase activity in HEK-293T (**B**) and HepG2 (**C**–**F**) cells, compared to the empty vector. Mutant allele decreases this enhancer effect in both cell lines. Treatments influence the luciferase activity regardless of the alleles. The *p*-values of the luciferase experiments (**B**–**F**) were calculated using the Welch *t*-test. The stars represent significance at different levels: * *p* < 0.05, ** *p* < 0.01, **** *p* < 0.0001. In the case of the treatments, data were normalized to the results of the “empty” vector. wt(C): wild-type allele; mt(T): mutant allele; TK: “empty” vector without insert; NC: negative control (random sequence); ns: not significant; AU: arbitrary unit.

The rs841847 intronic variant is in a suggested enhancer region (Enh-2) of the SLC2A1/GLUT1 gene. In our experiments for measuring the red cell membrane expression of the GLUT1 membrane protein, we found that this expression was significantly lower in the presence of the mutant (A) allele (Figure 3A, *p* = 0.0178). This finding may suggest that rs841847 decreases the enhancer activity on GLUT1 expression in the erythroid cells. 

When studying the luciferase expression by using the wild-type or mutant allele containing DNA constructs, we found that the insert had a significant enhancer/promoter effect both in HEK-293T and HepG2 cells (Figure 3B,C). Interestingly, in both cell types, the luciferase activity was significantly lower in the presence of the mutant allele (HEK-293T: *p* = 0.0039, Figure 3B; HepG2: *p* = 0.0243, Figure 3C). These results are consistent with the expression level of GLUT1 in the red cell membrane. In our experiments, examining the effects of various incubation conditions on luciferase expression in the HepG2 cell (Figure 3D–F), we found effects independent of the variants.

**Figure 4:**
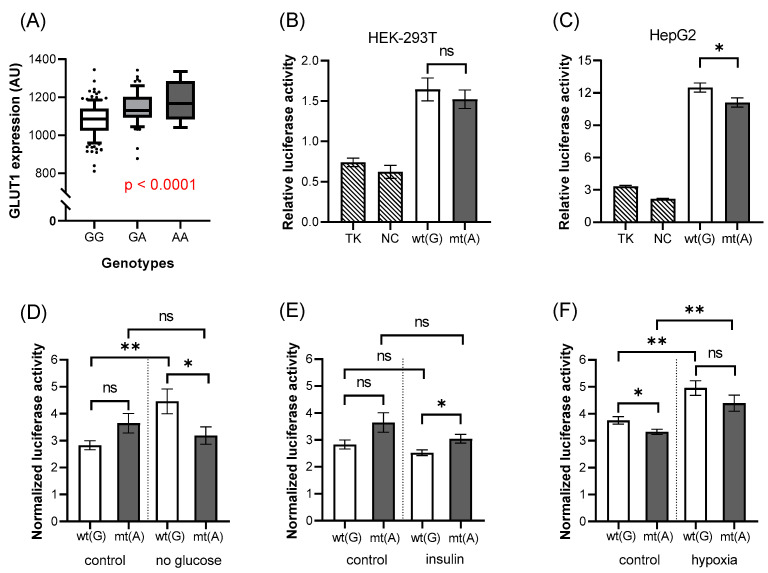
The effects of rs841848 on GLUT1 expression and luciferase activity. The association between the expression of the GLUT1 membrane protein and the genotypes of the variant (**A**). Each boxplot represents samples with different genotypes, in the range of 10–90 percentile, while dots represent samples out of range. This insert has increased luciferase activity in HEK-293T (**B**) and HepG2 (**C**–**F**) cells, compared to the empty vector but the mutant allele decreases this enhancer effect in HepG2 cells (**C**). In FBS-free medium (**D**,**E**), the opposite result could be seen. Wild-type allele has a stimulating effect on luciferase expression under low concentrations of glucose. Hypoxia increased luciferase expression, regardless of the alleles. The *p*-values of the luciferase experiments (**B**–**F**) were calculated using the Welch *t*-test. The stars represent significance at different levels: * *p* < 0.05, ** *p* < 0.01. In the case of the treatments, data were normalized to the results of the “empty” vector. Stars representing the strength of the significance; wt(G): wild-type allele; mt(A): mutant allele; TK: “empty” vector without insert; NC: negative control (random sequence); ns: not significant; AU: arbitrary unit.

The rs841848 intronic variant is in the same putative enhancer region (Enh2) as the rs841847 variant, yet there is no genetic coupling between these two SNPs (see above). In our experiments, we found that the expression level of the red cell GLUT1 membrane protein was significantly higher in the presence of the mutant (A) allele than in the presence of the wild-type allele (Figure 4A, *p* < 0.0001). Thus, the two relatively closely localized but independent SNPs had different effects on the red cell GLUT1 protein expression. 

In the luciferase expression studies (Figure 4B–F), we have seen the enhancer effects of these constructs as expected, both in the HEK-293T and the HepG2 cells. However, there was no appreciable difference in the luciferase expression caused by the presence of the minor allele in HEK-293T cells, and only a small decrease could be observed in HepG2 cells (HEK-293T: *p* = 0.514, Figure 4B, HepG2: *p* = 0.0354, Figure 4C). Interestingly, the presence of the FBS in the medium seems to have an influence on the outcome in this case. Comparing the control condition, the removal of the FBS for studying the glucose (Figure 4D) and insulin (Figure 4E) effects results in an increased luciferase expression in the presence of the minor SNP, while for the hypoxia treatment, in FBS containing medium, decreased luciferase expression was observed. Hypoxia (Figure 4F) produced a general increase in luciferase expression again, independent of the SNP.

**Figure 5:**
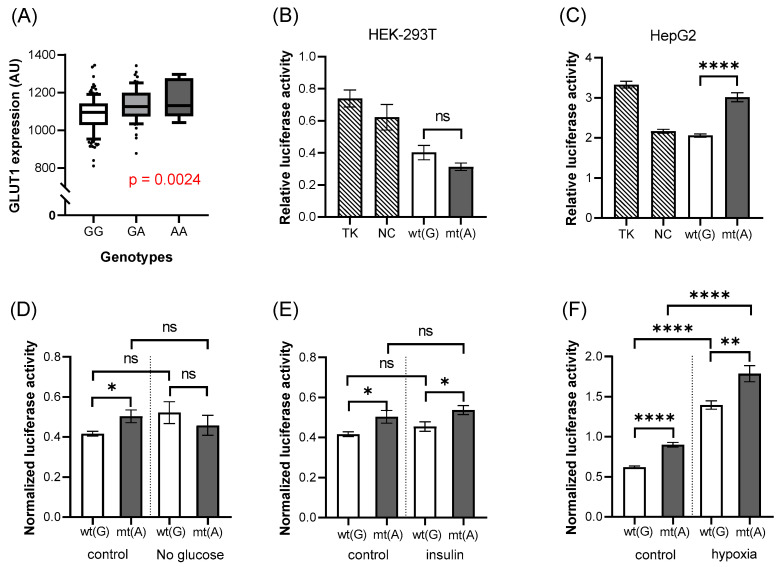
The effects of rs11537641 on GLUT1 expression and luciferase activity. The association between the expression of the GLUT1 membrane protein and the genotypes of the examined variant (**A**). Each boxplot represents samples with different genotypes, in the range of 10–90 percentile, while dots represent samples out of range. Luciferase activity in HEK-293T (**B**) and HepG2 (**C**–**F**) cells, under the effect of the insert containing the rs11537641 variant. This insert has decreased luciferase activity compared to the “empty” vector. In the presence of the mutant allele, luciferase activity does not show significant differences in HEK-293T (**B**) cells, but it shows in HepG2 (**C**) cells. The *p*-values of the luciferase experiments (**B**–**F**) were calculated using the Welch *t*-test. The stars represent significance at different levels: * *p* < 0.05, ** *p* < 0.01, **** *p* < 0.0001. Treatments (**D**–**F**) do not have a big influence on the results, although hypoxia increases luciferase expression, regardless of the alleles. In the case of the treatments, data were normalized to the results of the “empty” vector. Stars representing the strength of the significance; wt(G): wild-type allele; mt(A): mutant allele; TK: “empty” vector without insert; NC: negative control (random sequence); ns: not significant; AU: arbitrary unit.

The rs11537641 variant is located in exon 4 of the SLC2A1 gene. In the red cell GLUT1 expression studies, we found that the expression level was significantly higher in the presence of the mutant (A) allele (Figure 5A, *p* = 0.0024) than in the case of the wild-type allele. However, the luciferase expression studies indicated that this region has no enhancer activity, but rather slightly reduces luciferase expression driven by the TK promoter. The mutant allele had no significant effect on this suppressed expression in the HEK-293T cells, while in the HepG2 cells, it significantly increased the luciferase expression, thus reducing the suppressor effect of the construct (HEK-293T: *p* = 0.1015, Figure 5B, HepG2: *p* < 0.0001, Figure 5C). 

When we studied the effects of various incubation conditions (Figure 5D–F) in the HepG2 cells, we found no major effects of the SNPs on the luciferase expression. The only exception was the application of hypoxia (Figure 5F) which produced a big increase in luciferase expression, independent of the SNP.

According to our bioinformatics analysis, there are several predicted transcription factor (TF) binding sites that are altered in the presence of the tested SNPs (see Table 2). In the case of rs1385129, we found binding sites for EGR1, CTCF, and GCM1, in the presence of the wild-type allele. In contrast, a new binding site appears in the presence of the mutant allele for the ZNF281 transcriptional regulator. In the case of the mutant allele of rs841847, we found that binding sites for ARNT2, BHLHE40, BHLHE41, and ARNTL transcription factors disappeared, compared to the reference sequence. The wild-type allele of rs841848 influences binding sites for two important transcriptional regulators (ZNF460 and ZNF528), while the sequence with the mutant allele has new binding sites for STAT proteins (Stat1, Stat5a/b). In the case of the variant rs11537641, the sequence with the wild-type allele contains binding sites for the ZFP57 and Zfx transcriptional activators, while the mutant allele displays binding sites for ZBTB32, SREBF1, SREBF2, and FOXK1.

## 4. Discussion

Membrane proteins play a key role in numerous biological processes, and their quantitative changes are important in the development of various pathological conditions [30]. Several membrane proteins have already been suggested to provide important diagnostic biomarkers and drug targets [31]. The GLUT1 membrane protein is a key player in the cellular glucose uptake from the blood, especially in tissues directly depending on glucose metabolism. These include the human red cells, the brain, and kidney vascular tissues, as well as numerous malignant tissues [2,15,16]. The regulation of glucose metabolism in the endocrine pancreas also directly depends on the transporter-dependent sensing of blood glucose levels [32]. Specific mutations directly affecting GLUT1 protein expression and function are causing serious but rare disease conditions [7,8], while relatively small changes in the expression level of this protein in the key target tissues may have considerable effects in major complex metabolic diseases, including type 2 diabetes and Alzheimer’s disease [9,10].

In order to assess the relevance of genetic factors in complex diseases, GWA and case-control studies are widely performed and have become available to assess the potential connections of minor genetic variants with such diseases [33]. However, in most cases, the potential connection between direct protein expression levels and the presence of minor genetic variants is not specifically explored. In order to find such specific connections, in the present work we have performed a flow cytometry analysis to determine the expression level of GLUT1 in the red cell membrane and examined the role of the potentially connected SNPs by reporter expression studies.

Based on previous case-control studies [12,13,14] and our own sequencing experiments, we have selected four SNPs within the SLC2A1 gene, to be examined in detail (rs1385129, rs841847, rs841848, rs11537641) for the GLUT1 expression. These variants are in linkage disequilibrium with many other SNPs of the SLC2A1 gene, and two of them (rs1385129, rs841848) are parts of relatively large haplotypes. Thus, these variants can be regarded as lead SNPs, providing information for relatively large regulatory segments of this gene. The selected SNPs were analyzed in human volunteers together with the red cell expression levels of the GLUT1 protein. These experiments provided information not only on the effect of the tested SNP but also on the effect of the related haplotype. 

In order to analyze the direct modulation effect of the examined SNPs, we used luciferase reporter expression constructs of the surrounding genetic areas (see Supplementary Figure S2). Since the reporter plasmid constructs have to be relatively small, in these cases we searched only for the potential role of the examined SNPs, while other, connected SNPs may also contribute to the overall membrane GLUT1 expression levels seen in the red cells. As shown in the Section 3, we found significant associations between the red cell expression levels of the GLUT1, and the four SNPs examined. The presence of three of the variants (rs1385129, rs841848, and rs11537641) significantly increased red cell GLUT1 expression, while rs841847 reduced this membrane expression. We have also examined potential associations between these variants and T2DM, but—most probably due to the relatively low number of individuals examined—we did not find significant differences (see Supplementary Figure S5).

Our detailed reporter assays showed that the putative enhancer elements in intron 2, experimentally verified in mice, and containing rs841847 and rs841848 in humans, are also functional enhancers in the human SLC2A1 gene. In addition, in the luciferase assays, rs1385129, located in exon 2, also demonstrated a strong enhancer/promoter effect, while the region of rs11537641, located in exon 4, had a suppressing effect on general luciferase expression. 

Regarding the specific modulatory roles of these SNPs, the minor variant of rs1385129 slightly increased luciferase expression in the (renal derived) HEK-293T cells, while not in the (hepatoma derived) HepG2 model cells. This difference in these results suggests that this variant has a cell-specific effect. The human embryonic kidney-derived (HEK) cells may reflect kidney-type regulatory features and rs1385129 was previously described as a risk factor in diabetic nephropathy. Besides, in a previous study GLUT1 expression was increased in the renal glomerular cells, in a nephropathic rat model [34]. Taken together, this variant may contribute to DN through the regulation of GLUT1 expression. The presence of rs841847 significantly decreased this reporter expression in both cell types. The presence of the variant rs841848 in the reporter assays had no significant effect on luciferase expression in HEK-293T cells, but it showed an FBS-dependent effect in HepG2 cells. The variant rs11537641 had a significant increase in luciferase reporter expression only in the HepG2 cells. These data indicate a potentially tissue-dependent effect of the variants examined. 

When examining variable incubation conditions on luciferase expression in the HepG2 model cells, condition-dependent and, in some cases, SNP-dependent effects were seen. The effect of hypoxia increased luciferase expression in the case of all variants, corresponding to the observed regulation of the GLUT1 transporter in hypoxic conditions [35,36,37]. We found that the removal of the FBS or the changes in glucose concentrations variably modified the reporter expression responses compared to the standard incubation conditions. The direct effect of insulin addition did not appreciably modify the reporter expression responses in the HepG2 cells. 

The investigation of the transcription factor binding sites (TFBS) can be essential to understand the expression of these proteins, and the biological processes behind them as well. The bioinformatics analysis showed that there are several predicted transcription factor (TF) binding sites that are altered in the presence of the tested SNPs. In the case of rs1385129, we found three binding sites (EGR1, CTCF, and GCM1) in the presence of the wild-type (wt) allele. Since EGR1 has an important role in the response to glucose [38], insulin [39], and hypoxia [40], this change may have major regulatory consequences. In contrast, a new binding site appears in the presence of the mutant allele for the ZNF281 transcriptional regulator. In the case of rs841847, five TF binding sites disappeared compared to the wt allele, and these include ARNT2, potentially related to hypoxia responses [41]. The wt allele of rs841848 influences binding sites for 2 important transcriptional regulators (ZNF460 and ZNF528), while the sequence with the mutant allele has new binding sites for STAT proteins (Stat1, Stat5a, Stat5b), important in regulating cellular responses to insulin [42,43]. Interestingly, an even greater change is observed in the case of the variant rs11537641: the sequence with the wt allele contains binding sites for ZFP57 and Zfx, while the mutant allele displays 4 transcriptional factor binding sites (ZBTB32, SREBF1, SREBF2, and FOXK1). SREBF1 plays a role in several cellular responses, including those related to insulin receptors [44], while FOXK1 affects cellular glucose homeostasis [24].

Based on our current results and the previous data in the literature, we suggest that the minor SNPs and the connected haplotypes examined here may significantly modify the tissue-dependent expression of the GLUT1 protein. While the red cell expression data reflect the role of the genetic haplotypes, a caveat in our luciferase studies is that for a full evaluation of other SNP effects within the haplotypes, covering large genomic regions, a combination of numerous reporter studies would be required. The experimental verification of the predicted transcription factor or metabolite binding sites would also be required to further increase the knowledge of this complex genetic regulation.

## 5. Conclusions

This study presents a detailed analysis of the alterations of the GLUT1 expression and the potential genetic and regulatory background behind it. Our current results may initiate further detailed studies regarding the regulatory alterations and the role of specific transcription factors in these genomic regions and help the understanding of the background of GLUT1 expression changes in associated diseases. The presence of these SNPs may be an especially important factor in the complications of metabolic diseases, as previously observed in diabetic nephropathy.

## Figures and Tables

**Table 1:** Key data of the examined SNPs. G/C: wild-type allele (wt); A/T: mutant allele (mt); MAF: Minor Allele Frequency; Enh-2: enhancer 2.

SNP (Assay ID)	Alleles (wt > mt)	MAF (EUR)	Location
rs1385129 (C___1166185_1)	G > A	0.22	Exon 2
rs841847 (C___1166180_10)	C > T	0.25	Intron 2, Enh-2
rs841848 (C___8365112_10)	G > A	0.20	Intron 2, Enh-2
rs11537641 (C__30716206_10)	G > A	0.19	Exon 4

**Table 2:** Predicted transcription factor binding sites in the region of the examined SNPs. The table shows the binding sites (Sequence) of the transcription factors (TF name) affected by one of the alleles of the SNPs. The concerned DNA strand and the T2DM-specific function of the TFs are also described. The *p*-value refers to the binding probability of the TFs.

SNP ID	Allele	TF Name	Strand	Sequence	*p*-Value	Relevant Function
rs1385129	wild	EGR1	D	CTCCTCCCACGGCC	3.2 × 10^−4^	response to glucose, insulin, hypoxia
rs1385129	wild	CTCF	D	CGGCCAGCATGAGGCGACC	1.8 × 10^−4^	-
rs1385129	wild	GCM1	R	CATGCTGGCCG	2.4 × 10^−4^	-
rs1385129	mutant	Znf281	R	GCTGTGGGAGG	2.2 × 10^−4^	-
rs841847	wild	ARNT2	R	GTCCCGTGCA	2.6 × 10^−4^	response to hypoxia
rs841847	wild	BHLHE40	D	TGCACGGGAC	4.9 × 10^−4^	-
rs841847	wild	BHLHE40	R	GTCCCGTGCA	2.5 × 10^−4^	-
rs841847	wild	BHLHE41	R	GTCCCGTGCA	4.4 × 10^−4^	-
rs841847	wild	ARNTL	R	TGTCCCGTGC	1.1 × 10^−4^	-
rs841848	wild	ZNF528	R	CGGGAGGAAGGCTTTCC	2.5 × 10^−4^	-
rs841848	wild	ZNF460	D	AAGCCTTCCTCCCGAG	2.5 × 10^−4^	-
rs841848	mutant	STAT5a/STAT5b	D	CCTCCCAAGAA	1.3 × 10^−4^	response to insulin
rs841848	mutant	STAT1	R	GTTCTTGGGAG	4.9 × 10^−4^	cellular response to insulin
rs841848	mutant	Stat5a	D	CTCCCAAGAACC	2.7 × 10^−4^	response to insulin
rs11537641	wild	ZFP57	D	GTCAGGCCGCAGT	4.4 × 10^−4^	-
rs11537641	wild	Zfx	R	TGTACTGCGGCCTG	1.1 × 10^−4^	-
rs11537641	mutant	SREBF1	D	GTCAGGCCAC	2.8 × 10^−4^	insulin receptor signaling pathway, response to glucose
rs11537641	mutant	SREBF2	R	GTGGCCTGAC	1.2 × 10^−4^	-
rs11537641	mutant	ZBTB32	R	TGTACTGTGG	1.6 × 10^−4^	-
rs11537641	mutant	FOXK1	D	ACAGTACACACCGA	4.2 × 10^−4^	cellular glucose homeostasis

## Data Availability

The datasets generated and analyzed during the current study are available from the corresponding authors upon reasonable request.

## Supplementary Materials

The following supporting information can be downloaded at: https://www.mdpi.com/article/10.3390/biology11111669/s1, Figure S1: Haplotype blocks of rs1385129 and rs841848; Table S1: Linkage Disequilibrium between the 4 examined SNPs; Table S2: Sequencing results; Table S3: Sequencing primers; Table S4: Size of the inserts and sequence of the cloning primers; Figure S2: Vector Maps; Table S5: Clinical characteristics of the Control and T2DM groups; Figure S3: Mean levels of GLUT1 expression; Figure S4: GLUT1 expression levels are males and females; Figure S5: Genotype frequencies of the 4 SNPs in control and T2DM groups.
