# Supplementary material for: Genetic Modulation of the GLUT1 Transporter Expression—Potential Relevance in Complex Diseases
Source: Biology (Basel). 2022 Nov 16;11(11):1669. doi: 10.3390/biology11111669 (PMC9687623; doi:10.3390/biology11111669)
Supplement: Supplementary file 1 [file biology-11-01669-s001.zip › biology-2014222-supplementary.pdf]

Genetic modulation of the GLUT1 transporter expression
- potential relevance in complex diseases

Anna Kulin, Nóra Kucsma, Balázs Bohár, Botond Literáti-Nagy, László Korányi, Judit Cserepes, Anikó Somogyi, Balázs Sarkadi, Edit Szabó and György Várady

<u>Supplementary materials</u>

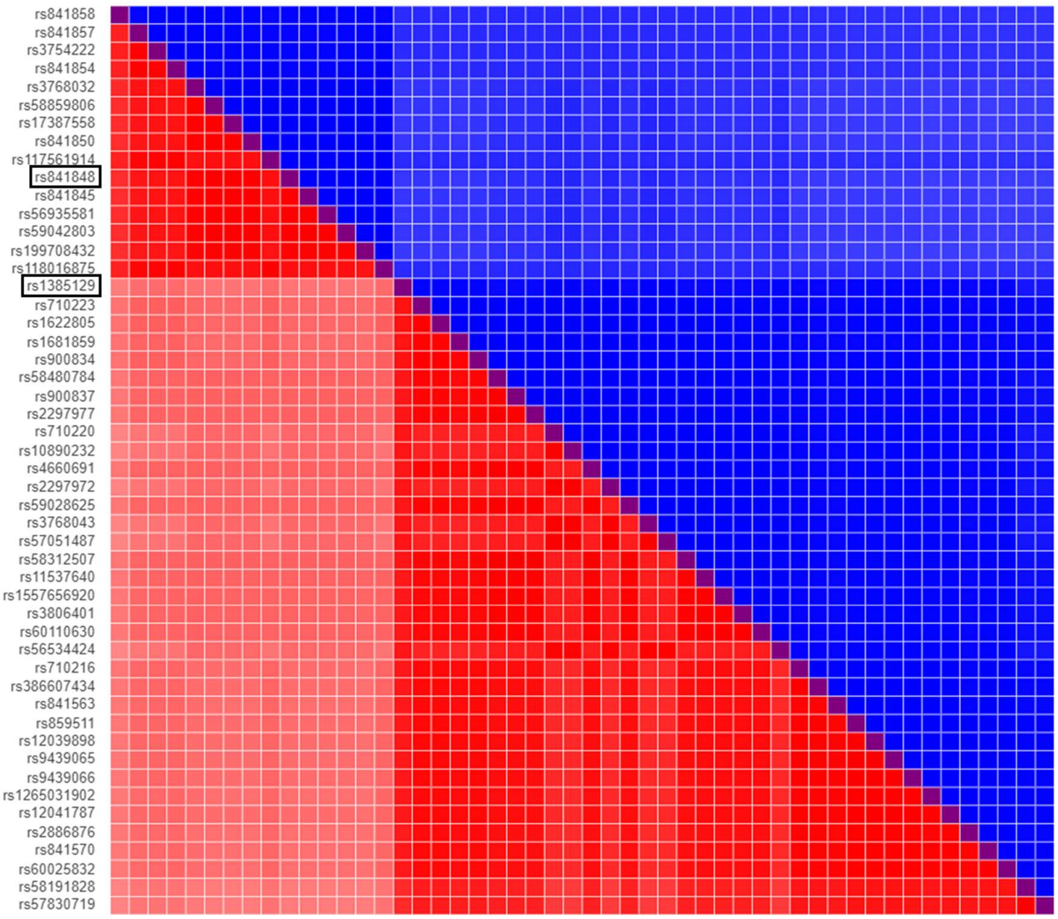

Figure S1. *Haplotype blocks of rs1385129 and rs841848. Linkage disequilibrium (LD) among the included SNPs is at least 0.8 (R2/D'). This LD matrix was made with LD Link site from European population; R square values (red shades), D prime values (blue shades).*

Table S1. *Linkeage Disequilibrum (R square) between the 4 examined SNPs.*

RS_number	rs1385129	rs841847	rs841848	rs11537641
rs1385129	1.0			
rs841847	0.09	1.0		
rs841848	0.55	0.08	1.0	
rs11537641	0.41	0.07	0.63	1.0

Table S2. *Sequencing results – Genetic variants in the SLC2A1 gene of samples with low, medium, and high RBC GLUT1 expression levels (Relative fluorescence, mean ± SD). From these polymorphisms we chose rs1385129 (previously described as risk factor in DN) and rs11537641. These two SNPs accumulate at higher GLUT1 expression levels. Variant rs2229682 is in linkage disequilibrium with rs11537641.*

	Relative fluorescence		SNPs			
**Sample**	**Mean**	**SD**	**rs1385129** MAF(EUR)=0.22	**rs11537641** MAF(EUR)=0.19	**rs2229682** MAF(EUR)=0.19	**rs11282849** MAF(EUR)=0.47
8	877.8	52.5	GG	GA	GA	-
5	926.8	160.7	GG	GG	GG	insAAATGGTGAG
13	1057.9	4.9	GA	GG	GG	-
33	1075.4	95.2	GA	GA	GA	-
26	1084.6	24.5	GG	GG	GG	-
29	1126.5	71.3	GA	GA	GA	-
37	1174.0	40.4	GG	GA	GA	-
24	1226.0	162.8	GA	GA	GA	insAAATGGTGAG
36	1296.4	18.4	AA	AA	AA	insAAATGGTGAG

Table S3. *Sequencing primers. Sequence of the designed forward and reverse primers of the exons in the SLC2A1 gene.*

Exons	Forward primer (5'→3')	Reverse primer (5'→3')
**1**	GTCCCAACGCAGAGAGAACG	CGGCCCGCTAGATCCGAA
**2**	AAAGACTGGTGTGGTGCCAA	AGAAAACTGGCTGGAGAGGC
**3-4**	TCTCTTAGGGAGGGGTGCAA	TGGTGCTGTGTTCTCTGGAC
**5-6**	AACAGGGCTCATGCTAGTGG	TTCTTCGGCAGAGGCGTATC
**7-8**	CAGTGTCCCTTCTGCCTGAG	GGAGCCAGAAAGTCAGACCC
**9**	ATAGCTCTGCTCTGGCCTCT	CCCTCAGTTTCCTCCTCAGC
**10**	TAGGTACAAGCGTGGTCTCAG	AGGTTTGGAAGTCTCATCCAGC

Table S4. *Size of the inserts cloned to the modified pGL3 vector and sequence of the designed forward and reverse primers (restriction enzyme cut sites are included and marked with italics). The inserts contain the minor allele only for the tested SNP.*

SNP	Insert size	Forward primer (5’→3’)	Reverse primer (5’→3’)
rs1385129	372 bp	ACT*GCTAGC*-AAAGACTGGTGTGGTGCCAA	AGTA*CTCGAG*-AGAAAACTGGCTGGAGAGGC
rs841847, rs841848	577 bp	GAC*GCTAGC*-CCATCTCCGTGCTGAGGTTT	ATGC*CTCGAG*-CCTCCCGGTAGACAGAGGAA
rs11537641	518 bp	CAT*GCTAGC*-GTGGGAGGTAGGGGAGACTT	ATCG*CTCGAG*-TGGTGCTGTGTTCTCTGGAC

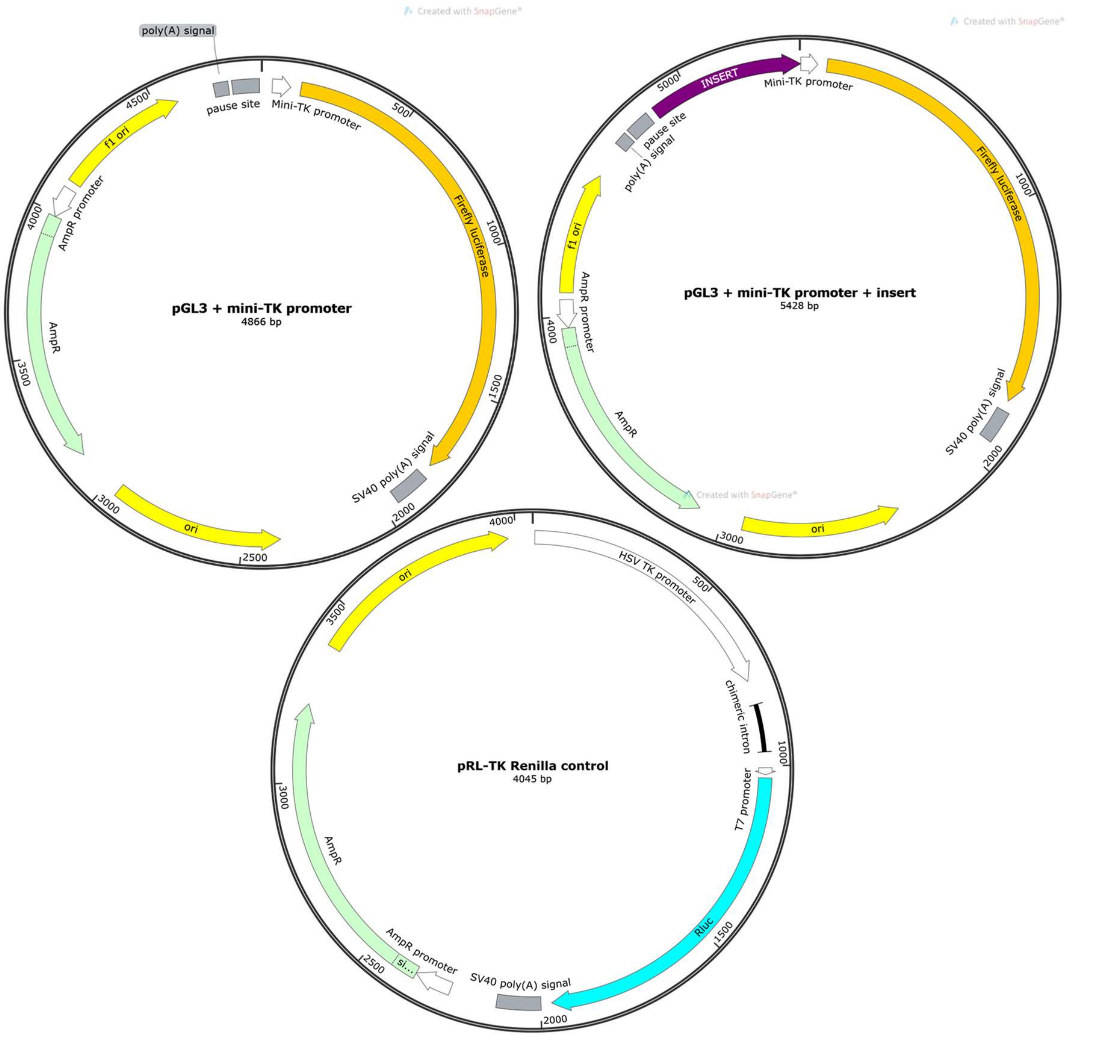

Figure S2. *Vector Map of the modified basic pGL3 vector and the inserts containing rs1385129, rs841847, rs841848 or rs11537641. Vector Map of the pRL-TK (Renilla luciferase vector with HSV-Thymidin kinase promoter).*

Table S5. *Clinical characteristics of the Control and T2DM groups. The values are expressed as means +/- SD. The p values were calculated by the Welch's t-test. N, Number; BMI, Body Mass Index; [a] Male to female; [b] values only for the samples from DRC. Here, due to the lack of data, values for the in-house volunteers are not presented.*

Variable	Control group	T2DM group	p-value
**N**	59	120	-
**Sex ratio (M/F)[a]**	1.0	0.8	-
**Age (years)**	60.8 ± 11.6	63.5 ± 9.2	0.1213
**BMI (kg/m^2)**	27.0 ± 4.7	30.3 ± 5.0	<0.0001
**HbA1c (%)**	5.3 ± 0.5	6.9 ± 1.3	<0.0001
**Glucose level (%)[b]**	5.0 ± 0.5	6.5 ± 2.1	<0.0001
**Insulin level (%)[b]**	7.8 ± 4.8	9.9 ± 4.8	0.0234
**HOMA index[b]**	1.8 ± 1.3	2.9 ± 1.8	0.0002

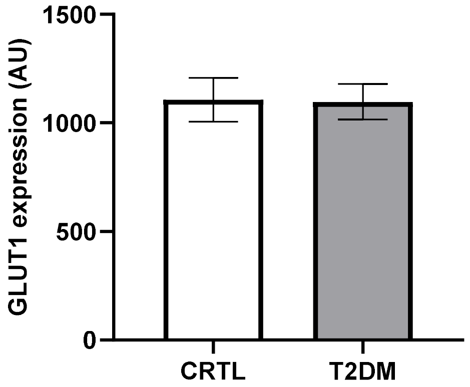

Figure S3. *Mean levels of GLUT1 expression in the control individuals (CTRL, n=87) and the T2DM patients (n=120).*

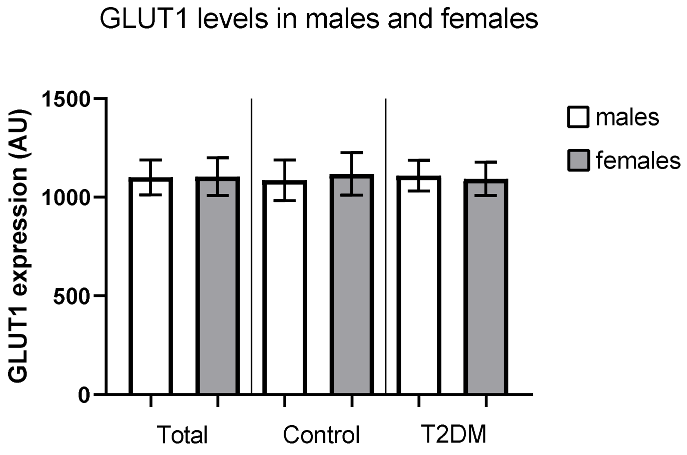

Figure S4. *GLUT1 expression levels in males and females. There was no difference in the GLUT1 expression levels between males and females (p=0.928). Among the control and patient groups significant differences were also not detectable (control: p=0.3085; T2DM: p=0.3321).*

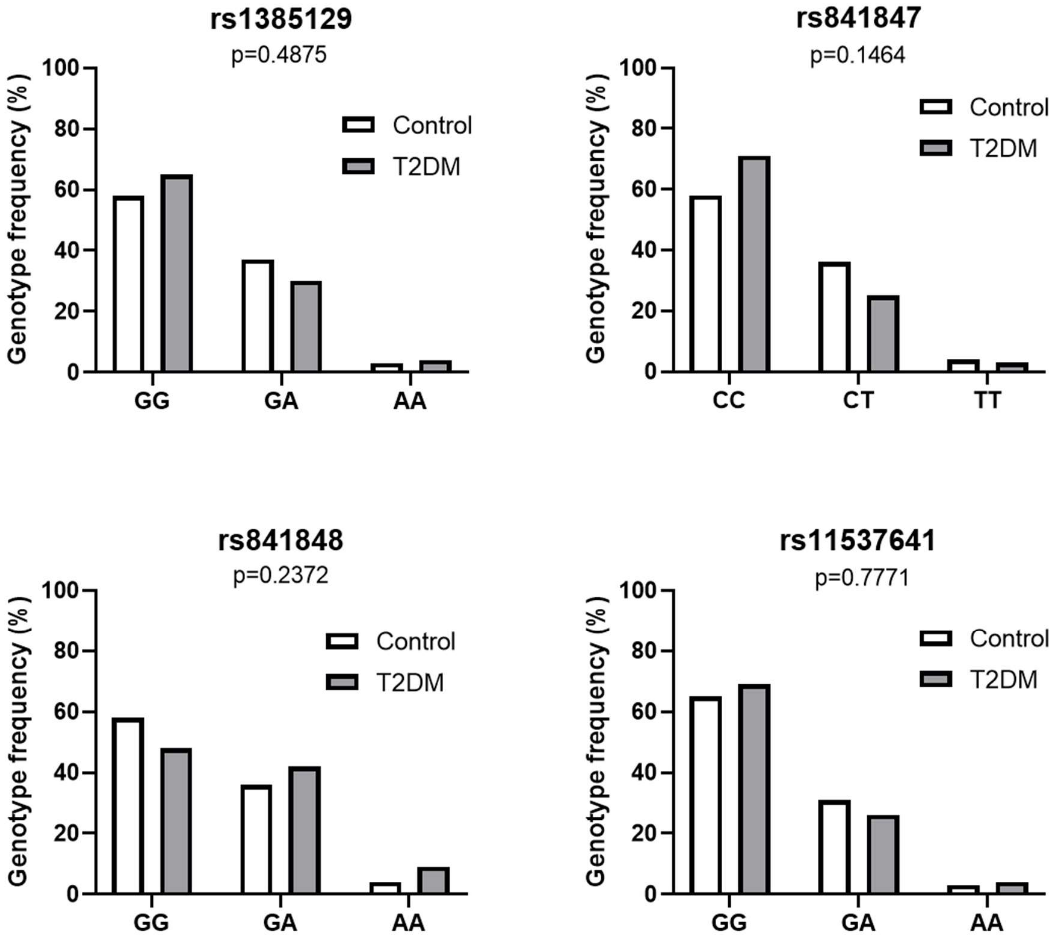

Figure S5. *Genotype frequencies of the 4 SNPs in the control (CTRL. n=87) and the T2DM groups (n=120). We did not find significant differences between the groups, although there is a tendency especially in case of rs841847 and rs841848. AU: arbitrary unit*
